# Efficacy of Corticosteroids Alone in the Eradication of Factor VIII Inhibitor in an Old Female with Idiopathic Acquired Haemophilia A: Description of a Case

**DOI:** 10.1155/2012/310730

**Published:** 2012-08-29

**Authors:** Francesco Girelli, Chiara Biasoli, Bruna Bassi, Franco Bagioni, Gabriele Bondi, Claudio Camporesi, Lucia Gardelli, Vincenzo Mazzeo, Maurizio Nizzoli

**Affiliations:** ^1^Specialistic Medicine Department, Internal Medicine Unit, Rheumatology Service, GB Morgagni Hospital, 47121 Forlì, Italy; ^2^Clinical Pathology Department, Immunotransfusional Unit, M Bufalini Hospital, Cesena, Italy; ^3^Immunotransfusional Unit, GB Morgagni Hospital, 47121 Forlì, Italy

## Abstract

Acquired haemophilia A (AHA) is a rare and serious disorder mainly affecting elderly patients. It is caused by the production of autoantibodies directed against coagulation factors; patients present with spontaneous bleeding, potentially fatal, in the absence of familial or personal history. Autoimmune disorders, infections, solid and hematologic tumors, and drugs are predisposing factors, but up to 50 percent of cases remain unexplained. The diagnosis of AHA is confirmed by specific laboratory tests; and the therapy is a clinical challenge, due to the fact that older patients are often affected by comorbidities. By passing agents may be used when persistent bleeding or haemodynamic instability is observed; corticosteroids, alone or with immunosuppressive therapy, are necessary to inhibit the production of the autoantibodies. We describe a case in which steroids in monotherapy successfully, safely, and persistently inhibited the production of anti-Factor VIII antibodies, in an old patient admitted after rheumatologic consult.

## 1. Case Report

We report a 75-year-old woman who was admitted to our internal medicine division with multiple spontaneous ecchymoses at lower limbs and right cruralgia associated with homolateral painful hip, lasting for one month, in the absence of personal or family previous bleeding or clotting disorders. Her past history included vitiligo, arterial hypertension, hypercholesterolemia, and remote uncomplicated posttraumatic fracture of the 12 vertebral body of the thoracic spine. On admission, laboratory investigations revealed severe anemia and thrombocytosis. Coagulation tests showed a prolonged aPTT and a normal PT-INR, Factor-VIII (FVIII) activity was 16%, and FVIII inhibitor was 16 Bethesda units (BU), leading to the diagnosis of AHA. Lupus anticoagulants (LAC) was negative; and Factors II, V, IX, X, XI, XII, and Von Willebrand were normal. Even in the absence of strong clinical suspicion, we performed some laboratory tests for specific infectious and autoimmune connective tissue diseases, that resulted normal: HCV, HBsAg, Parvovirus B19, EBV, CMV, Toxoplasma, ANA test, anti-DNA, ANCA, and anticardiolipin antibodies. The only autoimmune anti-ENA mild positivity, anti Ro-SSA, was not confirmed four months later; our patient had never reported sicca syndrome, and Schirmer test was negative, so the diagnosis of Sjögren syndrome couldn't be made. We performed an abdomen ultrasound (US) that revealed large muscle haematoma of the right iliopsoas. The patient was transfused with packed red-blood cells due to anemia and promptly treated with prednisone at dosage of 1 mg/Kg/day, strictly monitoring Hb levels and hematocrit. As no further hemorrhages were observed and aPTT was maintained normal, after one month the steroid dosage was gradually reduced. Six months after the discharge clinical and laboratory parameters were still stable with prednisone 6.5 mg/day.

## 2. Discussion

AHA is a very rare disease, potentially fatal, caused by the production of autoantibodies directed against circulating FVIII of coagulation, leading to susceptibility to spontaneous bleeding. AHA mostly affects elderly patients, without personal or familial history of bleeding, with an estimated annual incidence of 1.3–1.5/million per year. Another peak of incidence occurs in young women during the postpartum [1]. Associated conditions in elderly patients are solid and lymphoproliferative tumors [2–4], autoimmune disease [5] and drugs [6], in up to 50% of cases the etiology remains unexplained, as in our patient. To better exclude the presence of solid tumors, as indicated in the international recommendations [7], we performed a mammogram and a toraco-abdominal CT scan, resulting normal, in adjunction to the ultrasound that is considered an important diagnostic tool in revealing deep muscle hematomas [8].

AHA patients typically present with widespread spontaneous subcutaneous and muscular bleeds, the severity of which does not correlate with FVIII or its inhibitor levels. Hemorrhagic deaths (about 9–22% of cases) can occur within the first weeks from gastrointestinal or lung bleedings; fatal intracranial and retroperitoneal bleeding generally may occur later. Patients remain at risk of fatal bleeding until the inhibitor is eradicated, even in the presence of mild bleeding [9]. The diagnosis of AHA should be clinically suspected in every patient with spontaneous bleeding, without familial or personal history, and is confirmed by laboratory investigations revealing the typical prolonged aPTT with normal PT-INR, due to a deficiency of one of the intrinsic coagulation factors or the presence of an inhibitor (most commonly against FVIII). Also LAC positivity and heparin therapy, that are other causes of prolonged aPTT with normal INR, should be excluded [8].

Once the diagnosis has been achieved, therapy should be early initiated, aimed to arrest hemorrhage and to eradicate inhibiting factor. Acute and severe bleeding symptoms are considered conditions requiring the use of antihemorrhagic treatment with FVIII bypassing agents, irrespective of inhibitor titer and residual factor VIII activity. Frequent clinical assessment and strict monitoring of the haemoglobin level or hematocrit is a good indicator of significant bleeding [9]. 

To eradicate FVIII inhibitor corticosteroids (prednisone 1 mg/Kg/day) should be administered immediately following diagnosis; after a 4–6 weeks, total dose can be gradually reducted, based upon clinical status and aPTT. Our patient started prednisone at the dosage described one day after admission, when laboratory tests clearly conducted to the diagnosis of AHA, in direct collaboration, as recommended [9], with the immunotransfusional center of our hospital. One week after the first steroid administration we found, after an initial improvement, a new prolongation of aPTT, lasting only 24 hours; as clinical status and Hb level were maintained within normality, we continued to administer steroids alone, in refracted dose, until discharge. The total dose was then empirically reduced by about 10% every week. About twenty weeks after the first steroid administration, the patient was asymptomatic and laboratory tests showed normalization of aPTT and FVIII activity of 185% (see Table 1); the steroid dosage was further reduced to achieve a maintenance dose of 5 mg/day.

Steroids have been used in combination with oral cyclophosphamide (1.5–2.0 mg/Kg/day), for a maximum of six weeks [2], also as first line therapy, but no data showed superior overall survival with this strategy. Other cytotoxic or immunomodulatory drugs, as azathioprine [10, 11], cyclosporine-A [12], and, for prolonged periods, rituximab [4, 11, 13] have been successfully used. However, the risk/benefit ratio of these drugs [10] needs to be carefully evaluated in elderly “fragile” patients, often affected by comorbidities such as ischaemic heart disease, diabetes mellitus, and arterial hypertension. Moreover, cardiovascular preexistent conditions expose the patients to a thrombotic risk that the therapy of AHA may increase. In the case described, with the exception of arterial hypertension, the cardiovascular history was silent; in adjunction to this, the good initial response obtained with steroids and its stability suggested to continue with the same strategy. Even if it is known that the severity of bleeding does not correlate with FVIII or its inhibitor levels [9], care must be taken into account when FVIII activity increases, as a response to therapy, as we noted about sixty days after the first steroid administration, about the decision to give low dose aspirin. 

AHA should be suspected in any patient presenting with spontaneous bleeding, no familial or personal history, and prolongation of aPTT and normal PT-INR. Underling disease, especially tumors, need to be excluded. Treatment of AHA is a challenge, as mortality is still high and because of the possibility of late fatal bleeding, thrombotic events, and severe drug side effects.

## Figures and Tables

**Table 1:** Laboratory tests and prednisone dosage, during the period of observation.

	21/09/2011	01/10/2011	14/12/2011	10/02/2012
aPTT	Ratio > 8	>8		0.83
	90 seconds	>240 seconds		22
Factor VIII activity	1%	1%	271%	182%
Factor VIII inhibitor	16 Bethesda			
Hb	8.2 gr/dL,	9.2 gr/dL	9.4 gr/dL	13.5 gr/dL
Hct	25.8%		42%	42.3%
Prednisone	1 mg/Kg/day		0.5 mg/Kg/day	0.1 mg/Kg/day

aPTT: activated partial thromboplastin time, Ratio nv: 0.80–1.20. PT-INR: prothrombin time, nv 1.08. Factor VIII IL-TPO nv: 59–143%. Specific Factor VIII inhibitor, nv: <0.5 UB.
